# Hospital Abuse and the General Practitioner

**Published:** 1907-01-05

**Authors:** 


### HOSPITAL ABUSE AND THE GENERAL PRACTITIONER.

THE question of hospital abuse is one of vital importance to the general practitioner, for by the continual widening of the portals of the hospitals and the free admission to their administrations for all comers, the possible clientele of the private practitioner tends to be proportionately diminished, and his services are increasingly less indispensable to the public. There is also a tendency for the hospitals to move outwards from the Central London district and bring their services to the very doors of the large suburban areas. Again the increased facilities of locomotion by cheap tram services render a visit to a hospital pleasant and inexpensive. There is too in this vast suburban London an ever-increasing unstable proportion of the population, who live in a hand-to-mouth sort of way, have no permanent abode, have no local interests, and make no provision for sickness. They are thriftless and improvident, yet make a show of respectability and gentility. It is these people who seize every opportunity of getting anything for nothing, who figure conspicuously in the out-patient department of our voluntary hospitals, who support the large suburban theatres and music-halls, but will not support the local doctor so long as a tram-fare will provide them with free medical advice.

This free use of the hospitals not only injures the private practitioner by wthdrawing from him a large proportion of his clientele *in posse*, but its moral effect upon the mind of the public is of even greater consequence to him. Instead of the public being taught to estimate their health at a high value, and pay proportionately for its preservation and restoration, this free hospital aid tends to greatly depreciate in their eyes the services of the medical profession, and accordingly to underestimate the remuneration such services are entitled to receive.

If the present out-patient work of the hospitals were replaced by properly organised provident dispensaries, and if the out-patient department of the hospital were restricted to consultative work, specially sent from these dispensaries or from the private practitioner, the unsatisfactory and cursory examination of the patients by the medical officers of the institution could be ended.

It cannot be gainsaid that the hospital is of infinite service to the private practitioner by supplying, not only a consultant free of charge, but also the technical skill of the specialist and the full equipment of hospital appliances. It is in such cases that the hospital finds its sphere of greatest usefulness, and there can be no better judge of the suitability of a case for hospital treatment than the medical attendant. By a proper restriction of the use of the hospital by the public, a much larger proportion of the community would be compelled to make provision, in one way or another, for the fees of their own doctor, and it would rest with him to decide upon the appropriateness of any particular case for admission to a hospital.

Apart from the charitable relief of the suffering, the hospitals have also a great work to perform, both in the education of those entering the profession, and also in the furtherance of medical and scientific knowledge and research. For these purposes the hospitals require material in the sense of the suffering and the diseased. We are of opinion that the hospitals should not suffer from lack of material were they more dependent upon the general practitioner for the selection of cases, whereas the overcrowded condition of the out-patient department might be considerably relieved. This overcrowding consists largely of patients who are of no value to the teacher or the student, but are a considerable burden to the medical officers in charge and a tax upon the financial resources of the hospital. With the increasing difficulties of the general practitioner there is every probability that the number of students entering the profession will continue to diminish, and the unnecessary overcrowding of hospital out-patients, may force itself upon the attention of the hospital authorities by a lack of newly-qualified medical students to offer their free services as medical officers to these departments. There is no doubt that the hospital was never intended to compete with the private practitioner. It should rather be a source of continual assistance and support to him. We shall welcome the day when it can be shown that, by proper provisions and restrictions, the work of the hospital is made supplemental to that of the general practitioner.

